# Tonsillectomy reduces recurrence of IgA nephropathy in mesangial hypercellularity type categorized by the Oxford classification

**DOI:** 10.1007/s10157-015-1170-7

**Published:** 2015-09-28

**Authors:** Keita Hirano, Hoichi Amano, Tetsuya Kawamura, Kyoko Watanabe, Kentaro Koike, Akihiro Shimizu, Satoshi Endo, Nobuo Tsuboi, Hideo Okonogi, Yoichi Miyazaki, Masato Ikeda, Kazushige Hanaoka, Makoto Ogura, Satoru Komatsumoto, Takashi Yokoo

**Affiliations:** Division of Nephrology, Department of Internal Medicine, Ashikaga Red Cross Hospital, 284-1 Yobe-cho, Ashikaga, Tochigi 326-0843 Japan; Division of Nephrology and Hypertension, Department of Internal Medicine, Jikei University School of Medicine, Minato, Tokyo, Japan

**Keywords:** Corticosteroid therapy, Histological grade, IgA/C3 ratio, Mesangial hypercellularity, The Oxford classification, Tonsillectomy

## Abstract

**Background:**

In patients with IgA nephropathy (IgAN), recurrence after steroid pulse therapy is associated with reduced renal survival. However, the predictors of recurrence have not yet been clarified.

**Methods:**

All patients who received 6-month steroid pulse therapy from 2004 to 2010 in our four affiliated hospitals and achieved a reduction of proteinuria to <0.4 g/day 1 year after treatment were retrospectively evaluated. The primary outcome was proteinuria ≥1.0 g/day during follow-up or additional antiproteinuric therapy. Two histological classifications were evaluated, the Oxford Classification with a split system and Japanese histological grades (HGs) with a lumped system.

**Results:**

During a median follow-up of 3.4 years, 27 (26.7 %) of the 101 patients showed recurrence. Multivariate analysis showed that HG was the only significant predictor of recurrence, with HG 2+3+4 vs HG 1 having a hazard ratio of 7.38 (95 % confidence interval 1.52–133). Furthermore, in patients with mesangial hypercellularity according to the Oxford Classification, cumulative rate of recurrence-free survival was greater in patients with steroid therapy plus tonsillectomy compared with those who received steroid therapy alone (Log-rank test, *P* = 0.022). However, this association was not observed in patients without mesangial hypercellularity.

**Conclusions:**

HG is a novel predictor of recurrence after steroid pulse therapy in patients with IgAN. Moreover, the combination of steroid pulse therapy plus tonsillectomy may indicate a lower risk of recurrence in patients with mesangial hypercellularity, as defined by the Oxford Classification.

**Electronic supplementary material:**

The online version of this article (doi:10.1007/s10157-015-1170-7) contains supplementary material, which is available to authorized users.

## Introduction

IgA nephropathy (IgAN) is the most prevalent primary chronic glomerulonephritis worldwide which causes end-stage renal disease (ESRD) in up to 40 % of affected patients [1]. Baseline proteinuria is a predictive indicator of treatment requirement in IgAN patients. Proteinuria is attenuated by steroid therapy and renin–angiotensin–aldosterone system (RAAS) inhibitors, resulting in more favorable renal survival [2]. Some IgAN patients treated with these agents, however, may experience a recurrence of proteinuria after an initial response to therapy [3, 4]. In addition, higher levels of proteinuria during follow-up have been associated with subsequent end-stage renal disease [5, 6]. These findings suggest that recurrent proteinuria is a leading cause of an unfavorable renal prognosis. To date, however, few studies have identified the mechanisms by which baseline characteristics and initial treatment response or therapeutic options affect recurrent proteinuria in patients with IgAN. Identifying factors prognostic of recurrence and developing therapeutic strategies to reduce recurrence in patients with IgAN are therefore necessary.

Over the last decade, two histological classification systems for IgAN have been proposed: the Oxford Classification, a split system proposed by the International IgAN Network and the Renal Pathology Society [7], and histological grades (HGs), a lumped system proposed by the Japanese Study Group Special IgAN [8]. The associations of classification scores within these systems and renal survival have been evaluated. However, the association of each classification score with treatment response or risk of recurrence is still not well understood. Furthermore, a recent randomized controlled trial revealed that steroid pulse therapy plus pre-conditioning with tonsillectomy showed a greater antiproteinuric effect during the first year of follow-up than steroid pulse therapy alone [9]. However, the association between the combination of tonsillectomy plus steroid pulse therapy and subsequent recurrence remains to be elucidated.

Our recent retrospective study found that the achievement of <0.4 g/day proteinuria 1 year after 6-month steroid pulse therapy was significantly associated with favorable renal survival [10]. Endocapillary hypercellularity in the Oxford Classification and low HG were also significantly associated with better renal survival or response to steroid therapy. However, specific clinical and histological factors and treatment options associated with IgAN recurrence remain uncertain. This study therefore investigated the independent predictors of recurrence in patients with IgAN who achieved <0.4 g/day proteinuria 1 year after 6-month steroid therapy.

## Subjects and methods

### Study design and patients

A historical cohort design was used. A total of 169 patients received 6 months of steroid therapy between 2004 and 2010 in our four affiliated hospitals. Among these, 4 patients who were followed for less than 1 year and 24 patients who were recruited into a randomized controlled trial were excluded [9, 10]. Of the remaining 141 patients, 38 patients with ≥0.4 g/day urinary protein excretion volume (UPE) at 1 year were also excluded. Since achievement of <0.4 g/day UPE at 1 year predicted a favorable renal survival [10], it meant that this category indicated partial remission in the initial stage. In addition, two patients were also then excluded because of missing data on their IgA/C3 ratios. Ultimately, 101 patients who achieved <0.4 g/day UPE 1 year after 6 months of steroid therapy were analyzed for recurrence of IgAN. This study was conducted in accordance with the Declaration of Helsinki and approved by the Medical Ethics Committee of Jikei University School of Medicine (#24-104-6870).

### Definitions

Patients were evaluated every 6 months. The endpoint of recurrent proteinuria was defined as the first of two consecutive urine collections with ≥1 g/day UPE or the need for additional treatment, including steroids and/or RAAS inhibitors due to increasing UPE of less than 1 g/day, more than 1 year following the initiation of steroid therapy. A disappearance of proteinuria was defined as <0.3 g/day UPE, and a disappearance of hematuria was defined as urinary sediment containing <5 red blood cells (U-RBC) per high power field (hpf). Clinical remission was defined as the disappearance of both proteinuria and hematuria. Estimated glomerular filtration rate (eGFR) was calculated using the Japanese eGFR equation and was based on patient age, sex and serum creatinine concentration [11]. Renal dysfunction was defined as eGFR <60 ml/min/1.73 m^2^. Hypertension was defined as arterial blood pressure (BP) ≥130/80 mmHg or treatment with any antihypertensive medication, including RAAS inhibitors [12].

### Treatment

The protocol for steroid pulse therapy was essentially the same as that described earlier [3, 4], except that a half dose of intravenous methylprednisolone was used [9, 10]. In brief, patients received 0.5 g/day methylprednisolone intravenously for 3 consecutive days at 0, 2 and 4 months, and 0.5 mg/kg oral prednisolone every other day for 6 months. Some patients underwent tonsillectomy for chronic tonsillitis complicated with IgAN just before starting the 6-month steroid therapy. Patients were administered RAAS inhibitors and antiplatelet agents as needed.

### Histology

Of the 101 included patients, 91 underwent renal biopsy within 1 year prior to corticosteroid therapy. To assess the effects of steroid treatment on histopathological changes related to recurrence of IgAN, renal biopsy specimens were obtained from these 91 patients and routinely processed for light microscopy. Sections were stained with hematoxylin and eosin and periodic acid–Schiff, together with silver methenamine and Masson’s trichrome. Pathological variables included mesangial hypercellularity (M), endocapillary hypercellularity (E), segmental sclerosis (S), tubulointerstitial atrophy/fibrosis (T) and extracapillary hypercellularity (Ext), according to the Oxford Classification [7]. The HGs described recently by the Special Study Group on Progressive Glomerular Disease in Japan were also evaluated [8]. In brief, samples were divided into four histological grades, HG 1, HG 2, HG 3 and HG 4, which corresponded to <25, 25–49, 50–74 and ≥75 %, respectively, of the glomeruli exhibiting cellular or fibrocellular crescents, global sclerosis, segmental sclerosis or fibrous crescents.

### Statistical analyses

Normally distributed variables were expressed as means ± standard deviations (SD) and compared using the *t* test or one-way ANOVA. Nonparametric variables were expressed as medians and interquartile ranges (IQR) and compared using the Mann–Whitney U test, Kruskal–Wallis test, Spearman’s correlation or the Friedman test. Categorical variables were expressed in percentages and compared using the Chi-square test.

Event-free survival was analyzed using the Kaplan–Meier method. Factors independently prognostic of renal survival were also analyzed using multivariable Cox regression models. Variables with *P* values < 0.05 on univariate analysis were included in the multivariable model. The starting point of follow-up in survival analysis was defined as 1 year after steroid therapy. Receiver operating characteristic curves were drawn for each variable to determine the optimal cutoffs for predicting poorer outcomes. Different relevant multivariable models were tested in accordance with standard statistical rules. These results were expressed as hazard ratios (HRs) and 95 % confidence intervals (CIs).

*P* values <0.05 were considered statistically significant. All statistical analyses were performed using the JMP^®^ 9.0.0 (SAS Institute Inc.) and PASW statistics^®^ 18.0.0 (IBM) software programs.

## Results

### Clinical characteristics at the initiation of treatment and one year after treatment

The clinical characteristics of the 101 included patients at the start of steroid treatment and 1 year after treatment are presented in Table 1. Median initial proteinuria was 0.81 g/day, and mean initial eGFR was 75.1 ml/min/1.73 m^2^. Of the 101 patients, 33 (32.7 %) showed renal dysfunction, defined as an eGFR <60 ml/min/1.73 m^2^. Concurrent tonsillectomy was performed in 50 patients (49.5 %). In addition, 57 patients (56.4 %) had hypertension, defined as BP >130/80 mmHg or receiving any antihypertensive medications. One year after treatment, approximately half of the patients showed clinical remission, defined as the disappearance of both proteinuria and hematuria. Despite patients with clinical remission showing higher prevalence of U-RBC >30/hpf than those without clinical remission, there were no significant differences in other baseline characteristics between the two groups of patients.

**Table 1:** Clinical and histological characteristics of enrolled patients

Variables	Value
Clinical characteristics (*N* = 101)	
Start of treatment	
Age, years	34 (26–43)
Female	50 (49.5)
Tonsillectomy^a^	50 (49.5)
Hypertension^b^	57 (56.4)
UPE, g/day	0.81 (0.58–1.38)
U-RBC > 30/hpf	58 (57.4)
eGFR, ml/min/1.73 m^2^	75.1 ± 27.6
Renal dysfunction^c^	33 (32.7)
IgA, mg/dl	323 (253–397)
C3, mg/dl	100 (94–113)
IgA/C3 ratio	3.06 (2.50–3.92)
One year after the treatment	
UPE, g/day	0.18 (0.10–0.29)
Disappearance of proteinuria	79 (78.2)
Clinical remission	48 (47.5)
Histological characteristics (*N* = 91)^d^	
HG^e^	
HG1/HG2/HG3/HG4	24/49/18/0 (26.4/53.8/19.8/0)
Oxford classification^f^	
M0/M1	63/28 (69.2/30.8)
E0/E1	29/62 (31.9/68.1)
S0/S1	16/75 (17.6/82.4)
T0/T1/T2	75/13/3 (82.4/14.3/3.3)
Ext absent/Ext present	6/85 (6.6/93.4)

Values are presented as the medians (IQR), numbers (%) or mean ± SD

*N* number of patients, *UPE* urinary protein excretion, *U*-*RBC* urinary sediments of red blood cells, *eGFR* estimated glomerular filtration rate, *M* mesangial hypercellularity, *E* endocapillary hypercellularity, *S* segmental sclerosis, *T* tubulointerstitial atrophy/fibrosis, *Ext* extracapillary hypercellularity, *HG* histological grade

^a^Tonsillectomy as concurrent treatment

^b^Blood pressure >130/80 mmHg or receiving any antihypertensive medication

^c^eGFR <60 ml/min/1.73 m^2^

^d^Data from patients who underwent renal biopsy within one year before starting treatment

^e^[8]

^f^[7]

### Baseline pathological characteristics

Of the 101 enrolled patients, 91 (90.1 %) underwent a renal biopsy within 1 year prior to the initiation of steroid therapy. The pathological characteristics of these 91 patients are also summarized in Table 1. According to the Japanese classification system, HG 1 and HG 2 were observed in approximately 25 and 50 % of these patients, respectively. According to the Oxford Classification systems, approximately 30 % of the patients showed an M1 grade of mesangial hypercellularity.

### Clinical and histological factors predicting recurrent IgAN

During a median follow-up of 3.4 years (IQR: 1–4.8 years) after achieving <0.4 g/day proteinuria at 1 year, 27 patients (26.7 %) showed proteinuria ≥1.0 g/day or required additional treatment. Nineteen patients had ≥1.0 g/day UPE, whereas the other eight achieved <1.0 g/day UPE only with the addition of RAAS inhibitors (5 patients) or steroid therapy (3 patients). The estimated period of 75 % survival free from recurrence was 4.0 years (95 % CI: 2.0–5.1 years). As shown in Table 2, patients with recurrence showed significantly higher levels of proteinuria during follow-up except for one year after treatment, but only had more profound hematuria at the final observation compared to patients without recurrence. In contrast, patients with recurrence had worse renal function than those without recurrence throughout the whole follow-up period.

**Table 2:** Comparison of proteinuria, hematuria and eGFR between recurrence and non-recurrence, according to timing of measurement

Variables	Timing	Outcome		*P* value^a^
		Recurrence	Non-recurrence	
UPE, g/day*	0 year	1.07 (0.70–1.64)	0.79 (0.50–1.23)	0.039^#^
	1 year	0.20 (0.12–0.30)	0.18 (0.09–0.27)	>0.2
	Last observation	1.18 (1.00–1.40)	0.15 (0.10–0.30)	0.001^#^
Hematuria**	0 year	14 (51.9)	44 (59.5)	>0.2
	1 year	11 (40.7)	30 (40.5)	>0.2
	Last observation	22 (81.5)	22 (29.7)	0.001^#^
eGFR, ml/min/1.73 m^2^***	0 year	62.2 ± 21.5	79.8 ± 28.2	0.004^#^
	1 year	63.7 ± 21.1	81.0 ± 27.9	0.004^#^
	Last observation	58.9 ± 21.4	74.0 ± 23.8	0.005^#^

*UPE* urine protein excretion volume, *eGFR* estimated glomerular filtration rate

* Values are presented as median and interquartile range

** Values are presented as number of patients (%) with U-RBC ≥30/hpf at start of treatment and U-RBC ≥5/hpf at 1 year after treatment or last observation, respectively. *** Values are presented as mean and standard deviation.^a^ Recurrence vs Non-recurrence. ^#^ *P* < 0.05

Univariate analysis showed that, among the various clinical factors tested, tonsillectomy demonstrated a significant association with a reduced risk of recurrence, whereas hypertension, renal dysfunction and IgA/C3 ratio showed significant associations with an increased risk of recurrence (Table 3). Interestingly, the IgA/C3 ratio categorized by the receiver operating curve analysis was a better predictor of recurrence than IgA/C3 ratio as a continuous variable. Although risk of recurrence tended to be higher in males, this difference was not statistically significant. Improvement in none of the urinary abnormalities 1 year after steroid therapy was associated with the study outcome. Although the patients with recurrence showed higher level of urine protein excretion volume at start of treatment than those without recurrence (Table 2), the baseline proteinuria was not a significant predictor of the primary outcome.

**Table 3:** Clinical predictors of recurrent IgA nephropathy in univariate models

Clinical predictors	B	SE	*χ* ^2^	HR (95 %CI)	*P* value
Start of treatment					
Age, per 10 years	0.253	0.148	2.796	1.29 (0.96–1.71)	0.095
Male vs Female	0.359	0.200	3.355	2.05 (0.95–4.66)	0.067
Tonsillectomy^a,^*	−0.467	0.205	5.469	0.39 (0.17–0.86)	0.019^#^
Hypertension^b,^*	0.426	0.220	4.205	2.34 (1.04–5.98)	0.040^#^
UPE, per 1 g/day	0.209	0.204	0.960	1.23 (0.79–1.78)	>0.1
U-RBC > 30/hpf*	−0.221	0.386	0.327	0.54 (0.24–1.38)	>0.1
Renal dysfunction^c,^*	0.438	0.193	5.065	2.40 (1.12–5.18)	0.024^#^
IgA, per 10 mg/dl	0.030	0.017	2.957	1.03 (0.99–1.07)	0.086
C3, per 10 mg/dl	−0.137	0.137	1.037	0.87 (0.66–1.13)	>0.1
IgA/C3 ratio, per 1	0.335	0.152	4.398	1.40 (1.02–1.86)	0.036^#^
IgA/C3 ratio > 2.91^d,^*	0.635	0.248	8.215	3.56 (1.46–10.7)	0.004^#^
One year after the treatment					
UPE, per 0.1 g/day	0.174	0.189	0.851	1.19 (0.82–1.73)	>0.1
Disappearance of proteinuria*	−0.080	0.220	0.129	0.85 (0.38–2.18)	>0.1
Clinical remission*	−0.131	0.196	0.451	0.77 (0.35–1.65)	>0.1

*UPE* urinary protein excretion, *U-RBC* urinary sediments of red blood cells, *B* coefficient, *SE* standard error, *χ* ^2^ Chi-square likelihood ratio, *HR* hazard ratio, *CI* confidence interval

* Yes vs no

^#^ *P* < 0.05

^a^Tonsillectomy as concurrent treatment

^b^Blood pressure > 130/80 mmHg or receiving any antihypertensive medication

^c^Estimated glomerular filtration rate < 60 ml/min/1.73 m^2^

^d^Cutoff value determined using receiver operating curve analysis

**Fig. 1:**
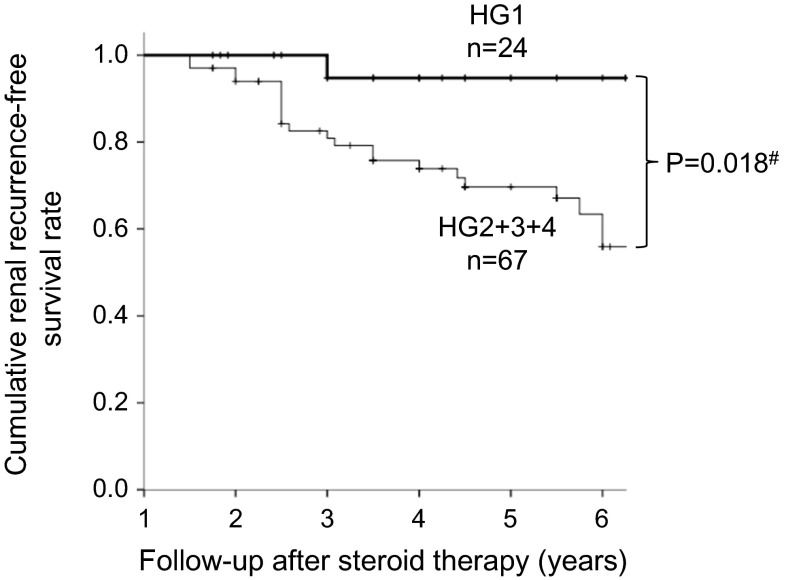
Kaplan–Meier analyses of recurrence-free renal survival in patients with HG1 and HG2+3+4

Univariate analysis showed that HG was the only histological factor significantly predictive of recurrence (Table 4), along with Kaplan–Meier analysis shown in Fig. 1 (Log-rank test, *P* value 0.018). Factors defined by the Oxford Classification, including mesangial hypercellularity (M0/M1) and endocapillary hypercellularity (E0/E1), segmental sclerosis (S0/S1) and tubulointerstitial fibrosis/atrophy (T0/T1+2) were not associated with recurrence.

**Table 4:** Histological predictors of recurrent IgA nephropathy in univariate models

Histological predictors	B	SE	χ^2^	HR (95 %CI)	*P* value
HG					
HG2+3+4 vs HG1	2.022	1.023	7.477	7.55 (1.59−135)	0.006^#^
Oxford classification					
M1 vs M0	0.063	0.228	0.076	1.13 (0.43−2.67)	>0.1
E1 vs E0	0.423	0.506	0.753	1.53 (0.61−4.63)	>0.1
S1 vs S0	0.380	0.370	1.298	2.14 (0.63−13.4)	>0.1
T1+2 vs T0	−0.024	0.253	0.009	0.95 (0.31−2.40)	>0.1
Ext present vs absent	−0.077	0.370	0.041	0.86 (0.25−5.37)	>0.1

*HG* histological grade, *M* mesangial hypercellularity, *E* endocapillary hypercellularity, *S* segmental sclerosis, *T* tubulointerstitial atrophy/fibrosis, *Ext* extracapillary hypercellularity, *B* coefficient, *SE* standard error, *χ* ^2^ Chi-square likelihood ratio, *HR* hazard ratio, *CI* confidence interval

^#^ *P* < 0.05

### Baseline HG as an independent predictor of recurrence

Multivariate analysis that included baseline factors significant in univariate analyses was performed to identify independent predictors of recurrence. This multivariate analysis showed that HG 2+3+4, compared with HG 1, was the sole significant independent predictor of risk of recurrence (HR: 7.38, 95 % CI: 1.52–133; Table 5). Tonsillectomy tended to be associated with a reduced risk of recurrence, whereas a higher IgA/C3 ratio tended to predict increased risk of recurrence.

**Table 5:** Multivariate Cox hazard model for predicting recurrent IgA nephropathy

Predictors	B	SE	*χ* ^2^	HR (95 % CI)	*P* value
HG2+3+4 vs HG1	1.999	1.030	6.956	7.38 (1.52–133)	0.008^#^
Tonsillectomy^a,^*	−0.439	0.231	3.814	0.42 (0.16–1.00)	0.051
IgA/C3 ratio > 2.91^b,^*	0.472	0.264	3.639	2.57 (0.98–8.05)	0.056
Hypertension^c,^*	0.112	0.252	0.201	1.25 (0.48–3.53)	>0.1
Renal dysfunction^d,^*	−0.026	0.237	0.012	0.95 (0.37–2.45)	>0.1

*B* coefficient, *SE* standard error, *χ* ^2^ Chi-square likelihood ratio, *HR* hazard ratio, *CI* confidence interval

* Yes vs no

^#^ *P* < 0.05

^a^Tonsillectomy as concurrent treatment

^b^Cutoff values determined using receiver operating curve analysis

^c^Blood pressure >130/80 mmHg or receiving any antihypertensive medication

^d^Estimated glomerular filtration rate <60 ml/min/1.73 m^2^

### Tonsillectomy as a predictor of recurrence in patients with mesangial hypercellularity

**Fig. 2:**
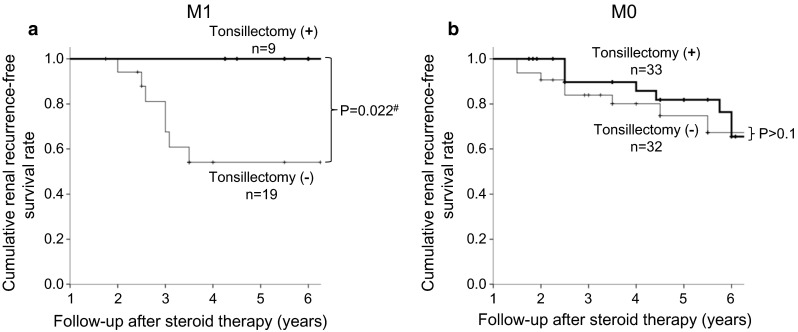
Kaplan–Meier analyses of recurrence-free renal survival in patients undergoing tonsillectomy plus steroid therapy and steroid therapy alone, stratified by mesangial hypercellularity score in the Oxford Classification. **a** Patients with mesangial hypercellularity (M1), as defined by the Oxford Classification. The combination of tonsillectomy plus steroid therapy significantly reduced the cumulative rate of recurrence compared with steroid therapy alone (*P* = 0.022, log-rank test). **b** Patients without mesangial hypercellularity (M0). There was no between group difference in cumulative rate of recurrence

Subgroup and stratified analyses of all baseline variables found only one significant interaction, between tonsillectomy plus steroid therapy (versus steroid therapy alone) and mesangial hypercellularity M1 (versus M0) (*P*_interaction_ = 0.015). When cumulative renal recurrence-free survival rate was compared in patients who underwent tonsillectomy and received steroid therapy with patients who received steroid therapy alone, a lower risk of recurrence was observed only in the patients with mesangial hypercellularity (M1) (Log-rank test, *P* = 0.022; Fig. 2a). Supplementary data from patients with M1 reveal no significant differences in clinical and histological characteristics between patients who underwent tonsillectomy and those who received steroid therapy alone. Conversely, among patients with M0, even though patients with tonsillectomy had some advantages at baseline, namely younger age and better renal function than those without tonsillectomy, anti-recurrent effect of tonsillectomy was not confirmed (Fig. 2b).

## Discussion

Recurrence remains a frequent and challenging problem in patients with IgAN [13]. The results of this large, multicenter cohort study suggested that HG was the only significant and independent predictor of recurrence in patients who achieved attenuated proteinuria following steroid therapy. Compared with patients with HG 1, those with HG 2+3+4 were at an approximately sevenfold increased risk for recurrence. The scoring system of HGs uses a lumped system consisting of five factors: cellular crescents, fibrocellular crescents, fibrous crescents, segmental sclerosis and global sclerosis. However, in our study, none of these individual pathological factors predicted recurrence, even on univariate analysis (data not shown). Therefore, the ability of HG to predict recurrent IgAN was the result of its lumped scoring system. Thus, HG is a predictor not only of renal survival [10], but of the recurrence of IgAN.

A stratified analysis for the prediction of recurrence showed the clinical impact of the Oxford Classification, which affected the response to tonsillectomy combined with steroid therapy. In particular, patients with mesangial hypercellularity (M1) had a lower risk of recurrence after tonsillectomy plus steroid therapy than after steroid therapy alone. Tonsillectomy combined with steroid therapy was also found to reduce the incidence of relapse (reappearance of urinary abnormalities) after clinical remission [14]. Moreover, recurrence was defined in this study mainly as ≥1.0 g/day UPE, since time-averaged proteinuria ≥1.0 g/day was found to predict poorer renal survival [5]. Furthermore, a recent study also showed that tonsillectomy plus steroid pulse therapy had a beneficial effect on the clinical course in IgAN patients with distinct M1 [15]. Although a combination of mesangial hypercellularity according to the Oxford Classification and tonsillectomy was associated with reduced recurrence in this study, the detailed mechanism by which tonsillectomy plus steroids affects recurrence remains unknown, indicating the need for future prospective studies.

This study also found that a high IgA/C3 ratio tended to indicate increased risk of recurrence. High IgA/C3 ratios were previously shown to be associated with adverse effects on renal survival. For example, serum IgA/C3 ratio was found to reflect the histological severity of IgAN and could therefore serve as a marker for IgAN progression [16]. Conversely, patients with a greater degree of IgAN progression had lower serum C3 and higher serum IgA levels [17]. However, IgA/C3 ratio had not been shown to predict the progressive course of IgAN after steroid therapy. Therefore, to our knowledge, this study is the first to report that a high baseline IgA/C3 ratio is predictive of IgAN recurrence.

Surprisingly, we found that urinary abnormalities, including proteinuria and hematuria, both at the start of treatment and 1 year after treatment, were not necessarily associated with renal outcomes. In addition, multivariate analysis showed that age, hypertension and poor renal function at baseline were not associated with these outcomes, although these factors had been mentioned to be general indicators of poor prognosis in patients with IgAN [18, 19]. The reason for these results may be derived from exclusion of patients who did not show an initial reduction in proteinuria and those with UPE ≥0.4 g/day at 1 year.

This study had several limitations. First, it did not include a control group of patients who received supportive therapy alone such as RAAS inhibitors. Therefore, the findings from this study may not be applicable to such a group of the patients. Second, the study population was small, the statistical power was low, and the follow-up period for evaluating outcomes was relatively short. Third, these results could not be adjusted for all confounding factors, since the study was retrospective in design and the indications for treatment were not controlled.

In conclusion, this study found that, in IgAN patients showing initial improvement in response to steroid therapy, a high HG in the Japanese classification system predicted recurrence. Moreover, tonsillectomy combined with steroid therapy was found to reduce the risk of recurrence, especially in patients with mesangial hypercellularity as defined by the Oxford Classification.

## Electronic supplementary material

Below is the link to the electronic supplementary material.

Supplementary material 1 (DOCX 25 kb)
