# Supplementary material for: Tonsillectomy reduces recurrence of IgA nephropathy in mesangial hypercellularity type categorized by the Oxford classification
Source: Clin Exp Nephrol. 2015 Sep 28;20:425–32. doi: 10.1007/s10157-015-1170-7 (PMC4891371; doi:10.1007/s10157-015-1170-7)
Supplement: Supplementary file 1 — Supplementary material 1 (DOCX 25 kb) [file 10157_2015_1170_MOESM1_ESM.docx]

| **Supplementary Table 1. Comparison of baseline characteristics between tonsillectomy (+) and (-) among patients with M1** | | | |
| --- | --- | --- | --- |
| Variables | Tonsillectomy | | P value |
|  | (+) | (-) |  |
| Number of patients | 9 | 19 |  |
| Age, years | 34 (26–43) | 37 (22–46) | >0.2 |
| Female | 4 (44.0) | 10 (52.6) | >0.2 |
| Hypertension ^a^ | 6 (66.7) | 13 (68.4) | >0.2 |
| UPE, g/day | 0.80 (0.62–1.50) | 0.94 (0.64–1.64) | >0.2 |
| U-RBC > 30/hpf | 5 (55.6) | 14 (73.7) | >0.2 |
| eGFR, ml/min/1.73m^2^ | 81.8 ± 39.1 | 75.6 ± 23.5 | >0.2 |
| IgA, mg/dl | 352 (216–481) | 306 (233–371) | >0.2 |
| C3, mg/dl | 102 (92–126) | 96 (94–108) | >0.2 |
| IgA/C3 ratio | 2.60 (2.50–4.95) | 3.05 (2.30–3.82) | >0.2 |
| **Note:** Values are presented as the medians (IQR), numbers (%) or means ± SD. ^a^ Blood pressure >130/80 mmHg or receiving any anti-hypertensive medication. **Abbreviations:** UPE, urinary protein excretion; U-RBC, urinary sediments of red blood cells; eGFR, estimated glomerular filtration rate. | | | |

| **Supplementary Table 2. Comparison of baseline characteristics between tonsillectomy (+) and (-) among patients with M0** | | | |
| --- | --- | --- | --- |
| Variables | Tonsillectomy | | P value |
|  | (+) | (-) |  |
| Number of patients | 33 | 30 |  |
| Age, years | 31 (20–39) | 36 (31–50) | 0.014 ^#^ |
| Female | 20 (60.6) | 12 (40.0) | >0.2 |
| Hypertension ^a^ | 15 (45.5) | 18 (60.0) | >0.2 |
| UPE, g/day | 0.71 (0.53–1.24) | 0.93 (0.58–1.42) | >0.2 |
| U-RBC > 30/hpf | 17 (51.5) | 18 (60.0) | >0.2 |
| eGFR, ml/min/1.73m^2^ | 83.7 ± 25.3 | 62.6 ± 27.5 | 0.002 ^#^ |
| IgA, mg/dl | 316 (256–359) | 326 (254–426) | >0.2 |
| C3, mg/dl | 103 (94–117) | 99 (94–106) | 0.144 |
| IgA/C3 ratio | 2.88 (2.47–3.70) | 3.21 (2.62–4.32) | 0.133 |
| **Note:** Values are presented as the medians (IQR), numbers (%) or means ± SD. ^a^ Blood pressure >130/80 mmHg or receiving any anti-hypertensive medication. **Abbreviations:** UPE, urinary protein excretion; U-RBC, urinary sediments of red blood cells; eGFR, estimated glomerular filtration rate. | | | |

| **Supplementary Table 3.** Predictors for recurrent proteinuria plus hematuria | | | | | |  |  |
| --- | --- | --- | --- | --- | --- | --- | --- |
| Predictors | Univariate | | Multivariate | | | |  |
|  | HR (95%CI) | P value | | HR (95%CI) | P value | | |
| HG2+3+4 vs HG1 | 6.81 (1.42-122 ) | 0.011 ^#^ | | 6.35 (1.25-116 ) | 0.022 ^#^ | | |
| Tonsillectomy * | 0.44 (0.18-0.99) | 0.049 ^#^ | | 0.44 (0.16-1.12) | 0.086 | | |
| IgA/C3 ratio>2.91 * | 2.91 (1.17-8.79) | 0.020 ^#^ | | 2.10 (0.79-6.58) | 0.141 | | |
| eGFR, per 10 ml/min | 0.84 (0.70-0.98) | 0.028 ^#^ | | 0.96 (0.79-1.15) | >0.2 | | |
| **Note** * Yes vs No. ^#^ P<0.05. **Abbreviations:** HG, histological grade; eGFR, estimated glomerular filtration rate; HR, hazard ratio; CI, confidence interval. | | | | | |  |  |

| **Supplementary Table 4.** Univariate analyses by predictors for increasing UPE >1.0 g/day or additional therapy with increasing UPE <1.0 g/day | | | | |
| --- | --- | --- | --- | --- |
| Predictors | Definition of outcome | | | |
|  | UPE>1g/day | | Addition of RAAS inhibitors or steroids  with increasing UPE | |
|  | HR (95%CI) | P value | HR (95%CI) | P value |
| HG2+3+4 vs HG1 | 5.16 (1.04-93.4) | 0.044 ^#^ | ND | ND |
| Tonsillectomy ^a,^* | 0.40 (0.15-1.02) | 0.056 | 0.36 (0.07-1.53) | 0.165 |
| IgA/C3 ratio>2.91 ^b,^ * | 4.31 (1.44-18.6) | 0.007 ^#^ | 3.05 (0.70-20.8) | 0.143 |
| Hypertension ^c,^ * | 4.66 (1.55-20.1) | 0.005 ^#^ | 0.93 (0.22-3.93) | 0.917 |
| Renal dysfunction ^d,^ * | 4.79 (1.89-13.7) | 0.001 ^#^ | 0.38 (0.02-2.16) | 0.316 |
| **Note:** ^a^ Tonsillectomy as concurrent treatment. ^b^ Cut-off values determined using receiver operating curve analysis. ^c^ Blood pressure >130/80 mmHg or receiving any anti-hypertensive medication. ^d^ Estimated glomerular filtration rate < 60ml/min/1.73m2. * Yes vs No. ^#^ P<0.05. **Abbreviations:** HR, hazard ratio; CI, confidence interval. | | | | |

| **Supplementary Table 5.** Distribution of MEST score according to HG | | | |
| --- | --- | --- | --- |
| Oxford classification | HG | | P value |
|  | 1 | 2+3+4 |  |
|  | (N=24) | (N=67) |  |
| M1 | 9 (37.5) | 19 (28.4) | >0.2 |
| E1 | 14 (58.3) | 48 (71.6) | >0.2 |
| S1 | 15 (62.5) | 60 (89.6) | 0.005 ^#^ |
| T1+2 | 0 ( 0.0) | 16 (23.9) | 0.001 ^#^ |
| **Note;** Values are presented as numbers (%). **Abbreviation;** ^#^ P<0.05 | | | |
